# Correction: Green removal and waste valorization of ciprofloxacin from water using zinc–iron LDH–chia seed biocomposites: integrated adsorption, computational modeling, and electrochemical conversion

**DOI:** 10.1039/d5ra90120k

**Published:** 2025-10-27

**Authors:** Samar M. Mahgoub, Hassan A. Rudayni, Ahmed A. Allam, Sulaiman A. Alsalamah, Asmaa Elrafey, Rania Abdelazeem, Amna A. Kotp, Mahmoud M. Abdelsatar, Rami Shafei, Rehab Mahmoud

**Affiliations:** a Materials Science and Nanotechnology Department, Faculty of Postgraduate Studies for Advanced Sciences, Beni-Suef University Egypt; b Department of Biology, College of Science, Imam Mohammad Ibn Saud Islamic University (IMSIU) Riyadh 11623 Saudi Arabia HARUDAYNI@imamu.edu.sa SAAlsalamah@imamu.edu.sa; c Environmental Science and Industrial Development Department, Faculty of Postgraduate Studies for Advanced Sciences, Beni-Suef University Beni-Suef Egypt asmaa_elrafey@hotmail.com raniashaban333@gmail.com; d Department of Geology, Faculty of Science, Beni-Suef University Beni-Suef Egypt mahmoud.abdelstar.1997@gmail.com; e Chemistry Department, Faculty of Science, Beni-Suef University Beni-Suef 62511 Egypt rehabkhaled@science.bsu.edu.eg abdelaty.mohamed@science.bsu.edu.eg

## Abstract

Correction for ‘Green removal and waste valorization of ciprofloxacin from water using zinc–iron LDH–chia seed biocomposites: integrated adsorption, computational modeling, and electrochemical conversion’ by Samar M. Mahgoub *et al.*, *RSC Adv.*, 2025, **15**, 37705–37726, https://doi.org/10.1039/D5RA06018D.

The authors regret that Rami Shafei’s name was shown incorrectly in the original manuscript and that one of the affiliations (affiliation *b*) was also shown incorrectly. The affiliation should have been shown as: ^*b*^*Department of Biology, College of Science, Imam Mohammad Ibn Saud Islamic University (IMSIU), Riyadh 11623, Saudi Arabia*. The corrected lists of authors and affiliations are as shown herein.

The Royal Society of Chemistry apologises for these errors and any consequent inconvenience to authors and readers.

